# Dynamic multilayer networks reveal mind wandering

**DOI:** 10.3389/fnins.2024.1421498

**Published:** 2024-11-14

**Authors:** Zhongming Xu, Shaohua Tang, Zengru Di, Zheng Li

**Affiliations:** ^1^International Academic Center of Complex Systems, Beijing Normal University, Zhuhai, China; ^2^School of Systems Science, Beijing Normal University, Beijing, China; ^3^Department of Psychology, Faculty of Arts and Sciences, Beijing Normal University, Zhuhai, China; ^4^Center for Cognition and Neuroergonomics, State Key Laboratory of Cognitive Neuroscience and Learning, Beijing Normal University, Zhuhai, China

**Keywords:** multiplex networks, electroencephalograph, mind wandering, video-learning, functional connectivity

## Abstract

**Introduction:**

Mind-wandering is a highly dynamic phenomenon involving frequent fluctuations in cognition. However, the dynamics of functional connectivity between brain regions during mind-wandering have not been extensively studied.

**Methods:**

We employed an analytical approach aimed at extracting recurring network states of multilayer networks built using amplitude envelope correlation and imaginary phase-locking value of delta, theta, alpha, beta, or gamma frequency band. These networks were constructed based on electroencephalograph (EEG) data collected while participants engaged in a video-learning task with mind-wandering and focused learning conditions. Recurring multilayer network states were defined via clustering based on overlapping node closeness centrality.

**Results:**

We observed similar multilayer network states across the five frequency bands. Furthermore, the transition patterns of network states were not entirely random. We also found significant differences in metrics that characterize the dynamics of multilayer network states between mind-wandering and focused learning. Finally, we designed a classification algorithm, based on a hidden Markov model using state sequences as input, that achieved a 0.888 mean area under the receiver operating characteristic curve for within-participant detection of mind-wandering.

**Discussion:**

Our approach offers a novel perspective on analyzing the dynamics of EEG data and shows potential application to mind-wandering detection.

## 1 Introduction

During concentrated learning of live or recorded lectures, the human mind is prone to turning inwards. This inherent mental process is often referred to as mind-wandering (MW). Generally, MW is defined by attention oriented away from an external task toward our internal, self-generated thoughts (Dong et al., 2021). Extended periods of MW correlate negatively with direct educational outcomes, manifesting as decreased information retention and comprehension (Risko et al., 2012; Szpunar et al., 2014). Additionally, MW may indirectly affect learning outcomes, potentially impairing note-taking abilities (Lindquist and McLean, 2011). However, our current understanding of the neural mechanisms of MW remains incomplete, and there is a lack of reliable and objective methods to accurately detect it (Dhindsa et al., 2019), which are critical for the development of strategies to mitigate the negative impact of MW.

In MW detection, task-related assessments (Zhang and Kumada, 2018) and physiological measures such as eye movement, pupillometry (Faber et al., 2018), heart rate, skin conductance, and synchronization between respiration and sensory pressure (Zheng et al., 2019) are commonly-used. Neural measures, including functional magnetic resonance imaging (fMRI) and electroencephalography (EEG), provide direct insights into this mental state (Dong et al., 2021). FMRI offers detailed spatial resolution, but with low temporal precision. Conversely, EEG captures brain activity at high temporal resolution and is cost-effective and wearable, but lacks the spatial accuracy of fMRI, especially for deep brain sources.

Recent studies have focused on using event-related potentials and spectral features to classify MW. A sustained attention task showed decreased P300 amplitude during MW (Smallwood et al., 2008), and other experimental studies support the idea that MW reduces cognitive resources for task processing (Kam and Handy, 2013; Kam et al., 2012; O'Connell et al., 2009). An experiment focusing on mindful breathing revealed lower occipital alpha and fronto-lateral beta power during MW compared to breath focus (Braboszcz and Delorme, 2011). Similar conclusions have been found in another study (van Son et al., 2019). Functional connectivity is an important feature in studying MW. Investigations using fMRI on MW suggest that it is associated with the default mode network (DMN) and the executive control network. The DMN is composed of brain regions that remain activation during rest and is linked to MW (Mason et al., 2007). A study found DMN to be active during self-reported instances of MW (Mooneyham and Schooler, 2013). However, DMN also exhibits heightened activity during purposeful internal thought, including future planning and episodic memory retrieval (Spreng et al., 2009; Buckner et al., 2008; Andrews-Hanna, 2012). This means activation of the DMN is not a specific indicator of MW.

MW is a highly dynamic process characterized by rapid fluctuations and spontaneity in thought, but the corresponding changes in functional connectivity networks over time are often overlooked (Konjedi and Maleeh, 2021; Christoff et al., 2016). Considering the dynamics during MW is thus crucial for better understanding and detection. Compared to fMRI, EEG offers higher temporal resolution, enabling better capture of the dynamical aspects of brain functional connectivity. However, to our knowledge, there is currently no EEG functional connectivity analysis for MW (Kam et al., 2022). Traditional brain network analysis often overlooks critical information, such as frequency or time domain relationships. Multilayer networks can integrate multiple data sources, capturing connectivity across different frequency bands, time scales, and variations in connectivity during different tasks. For example, in epilepsy, multilayer EEG networks revealed less variable activity during absence seizures (Leitgeb et al., 2020). In stroke, they showed reduced global connectivity and robustness (Hao et al., 2024); while in Alzheimer's disease, multiplex networks indicated disrupted brain activity and achieved high classification accuracy (Cai et al., 2020). A time-window-based multilayer model also enhanced understanding of brain dynamics during driving (Chang et al., 2022). These examples highlight how multilayer networks improve insights into brain function and cognition. Considering the above, our study will analyze the dynamic characteristics of multilayer functional connectivity networks built from EEG during MW.

In this study, we developed a multilayer network analysis framework that integrates different functional connection definitions, with network nodes representing electrodes. To sidestep the difficulties associated with selecting window size and sliding step size in traditional sliding window data segmentation methods (Xu et al., 2023), we improved our previous segmentation algorithm to apply it to multilayer networks. Our multilayer network features two layers: one using amplitude envelope correlation (AEC) for intra-layer edge weights and the other employing imaginary phase-locking value (IPLV) for intra-layer edge weights. In this study we used only two layers, but our method is scalable to more layers. The network is a weighted undirected multiplex network for each time segment. Quantified by overlapping node closeness centrality vectors, we clustered these networks into four states, each representing a recurring motif, and discretized EEG segments into time series of network state labels. Finally, we implemented a hidden Markov model to classify presence of MW based on these state sequences, assessing the effectiveness of our multilayer analysis.

The remainder of the article is organized as follows. We present a detailed description of the proposed method in Section. 2. We present results in Section 3. Finally, we discuss our work in Section 4 and conclude in Section 5.

## 2 Method

### 2.1 EEG data and pre-processing

#### 2.1.1 Dataset

In this work, we conducted a detailed analysis of our EEG dataset that has been previously published (Tang et al., 2023). For more comprehensive methodological details, see that article; a brief summary is below.

#### 2.1.2 Participants and task design

A total of 14 healthy participants (six females and eight males; average age 23.36 ± 4.75 years) were engaged in the study. The two distinct experimental conditions were: the focused learning (FL) condition, during which the two most highly-rated (for interest) lecture videos chosen by each participant was shown to him or her; and the future planning condition, during which the two least interesting videos were shown. In the future planning condition, in which participants engaged in personal future planning cued by images (previously collected from them) displayed on screen before the lecture video. Following each video, participants provided feedback through rating scales, assessing their estimates of percentage of time focused on the video and percentage of time in intentional and unintentional mind-wandering. In this study, the mental state during the future planning condition is assumed to be MW (and called the MW condition hereafter), while that in the FL condition is assumed to be non-MW. During the video, participants could press a key to indicate deviation from the task in each condition, data thus marked are not analyzed here.

#### 2.1.3 EEG acquisition and pre-processing

The EEG data analyzed here were collected using an 8-channel system (Yiwu Jielian Electronic Technology Co., Ltd, China) at a sampling rate of 1,000 Hz. The low channel count was intended to make the system more easily deployable. Electrodes were positioned at F3, F4, T3, C3, C4, T4, O1, and O2 according to the International 10-20 EEG system, with the reference electrode at CZ. The raw data underwent high-pass filtering at 1 Hz, down-sampling to 256 Hz, and notch filtering at 48–52 Hz. To mitigate noise interference, we implemented artifact subspace reconstruction (ASR) using the clean raw data plugin of EEGLab. Pre-task resting-state signals served as calibration data, with artifacts being either removed or corrected based on specific parameters (“BurstCriterion”=30 and “WindowCriterion” = 0.3) (Kothe and Jung, 2016). Additionally, we employed an amplitude threshold-based method to further detect and eliminate noise-contaminated time segments. Subsequently, EEG signals were band-pass filtered into delta (1–4 Hz), theta (4–8 Hz), alpha (8–13 Hz), beta (13–30 Hz), and gamma (30–80 Hz) frequency bands using windowed-Hamming finite impulse response filters, using the default filtering method and settings of MNE-Python (Gramfort et al., 2013).

### 2.2 Multilayer network construction

#### 2.2.1 Design of multilayer networks

**Figure 1:**
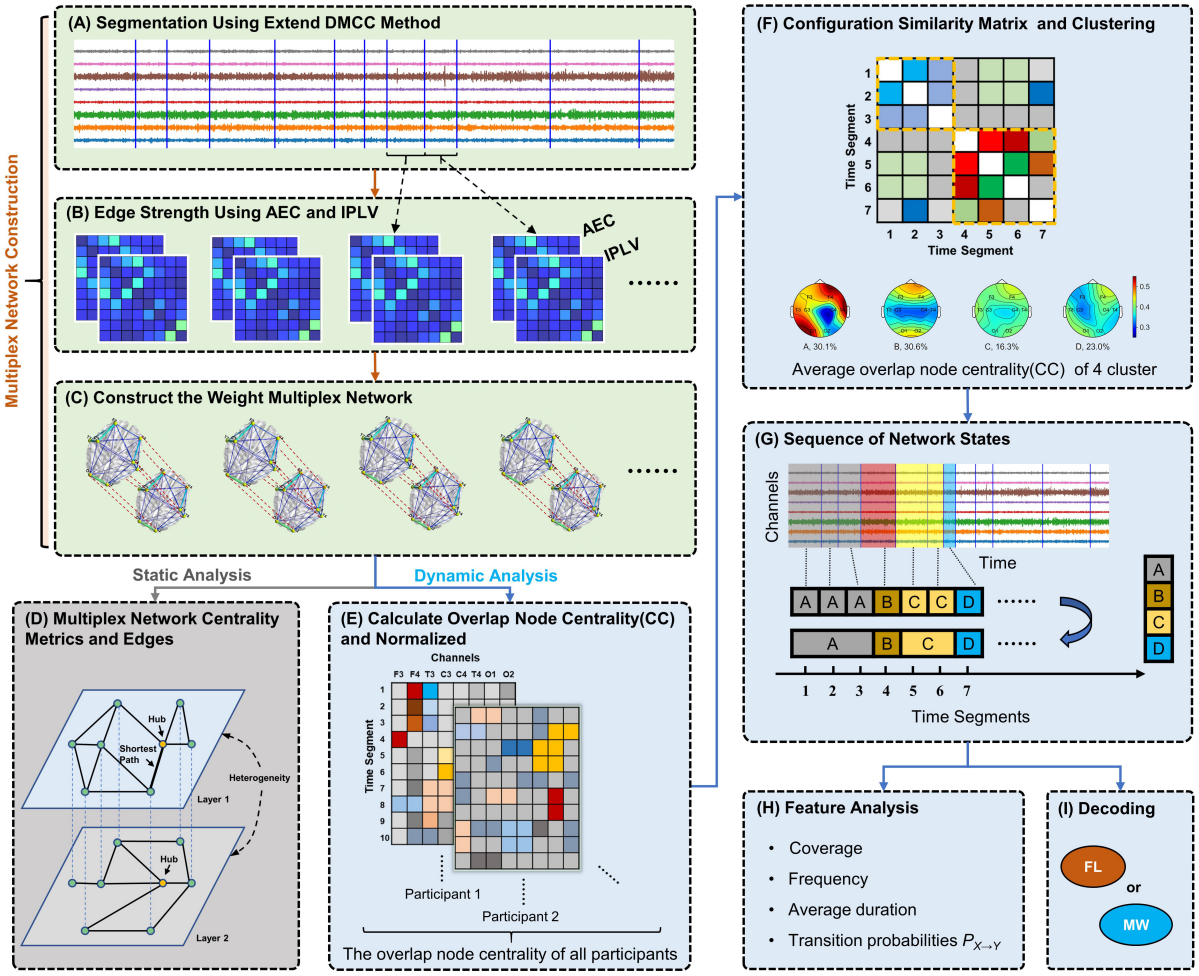
Diagram of analytical methods. **(A)** Segmentation of 8-channel EEG data based on changes in network structure. **(B)** Construction of functional connectivity networks for each segment using AEC and IPLV for the first and second layer, respectively. **(C)** Weighted multiplex networks are formed. Connection thickness in visualization indicates edge weight. **(D)** Analysis of static network topology (one segment). **(E)** Calculation of overlap node closeness centrality for each node. **(F)** Clustering of networks (segments) by their node centrality vectors using the Louvain algorithm, producing clusters representing typical recurring network states. **(G)** Discretization of EEG data into a network state sequence based on clustering results. **(H)** Computing and analysis of various dynamical features (statistics of state sequences). **(I)** Classification of network state sequences as mind-wandering or focused learning via a hidden Markov model.

In this study, we construct weighted multilayer networks which integrate two types of functional connectivity. Taking a segment of EEG data as an example (see Figure 1A), we assessed the functional connectivity between each pair of EEG channels using AEC and separately IPLV. Thus, two 8 × 8 connectivity matrices are obtained, as illustrated in Figure 1B. We construct a two-layer weighted network (see Figure 1C), where nodes of the network correspond to channels.

The edge weights in the first layer are determined by AEC. The computational procedure for AEC is delineated by the following rules (Bruns et al., 2000; Zamm et al., 2018): amplitude envelopes were generated via Hilbert transform of the EEG data in the segment; Pearson correlations were then computed between all amplitude envelopes across all combinations of channel pairs. Finally, we calculated the absolute values of the correlation coefficients to prevent cancellations during averaging, as envelope correlations can be negative. Additionally, negative values lack practical significance in brain network analysis for our purposes.

Considering that the edges in the first layer are calculated from the perspective of amplitude, to minimize overlap with the information of the first layer, we chose IPLV to measure functional connectivity strength in the second layer. The calculation of IPLV is as follows (Sadaghiani et al., 2012):


(1) $$\begin{array}{l} IPLV=\frac{1}{N}|Im\left(\overset{N}{\underset{n=1}{\sum }}exp\left[i\left(\phi_{channel1}\left(n\right)-\phi_{channel2}\left(n\right)\right)\right]\right)|, \end{array}$$

where *N* is the total number of time points within an EEG data segment, and ∣·∣ denotes the complex modulus. The Hilbert transformation is used to calculate the analytic phase at time point *n* as ϕ_*channel*_*j*_(*n*) = *arctan*[*u*(*n*)/*v*(*n*)], where *v* is the real part of the analytic signal, *u* is the Hilbert transform or the imaginary part *Im*(·) of the analytic signal.

The two crucial questions for constructing multilayer networks are: (1) whether there are differences between interlayer and intralayer connections; (2) whether different layers hold distinct meaning. We set a one-to-one correspondence between nodes in different layers based on the position of EEG channels, and interlayer connections exclusively link a node to its corresponding node in the other layer. This type of multilayer network is also known as a weighted multiplex network (Boccaletti et al., 2014). The interlayer edge weights are unconstrained, that is, there is no traversal cost associated with interlayer edges. Considering the possibility of different scales for edge weights between layers, we normalize each edge *e*_*i*_ of the layer *i* in our multilayer network as follows:


(2) $$\begin{array}{l} e_{i}=\frac{e_{i}-r_{min,i}}{r_{max,i}-r_{min,i}}, \end{array}$$

where *r*_*min, i*_ and *r*_*max, i*_ represent the minimum and maximum values e of the edges of layer *i*.

#### 2.2.2 EEG segmentation

To avoid an EEG data segment containing more than one network motif, we segmented EEG based on network structure using the DMCC (distance measure/closeness centrality) variant of our previously published segmentation method (Xu et al., 2023). Our method was previously designed for single-layer networks, thus we here improve the method by comparing each layer between time windows, and we call the new variant the extend DMCC (EDMCC). Given two time periods *T*_1_ and *T*_2_, the extended procedure computes the following distance measure:


(3) $$\begin{array}{l} d\left(\text{C}\left(T_{1}\right),\text{C}\left(T_{2}\right)\right)=\sqrt{\overset{n}{\underset{i=1}{\sum }}{\left(\frac{1}{m}\overset{m}{\underset{l=1}{\sum }}c_{i}^{\left[l\right]}\left(T_{1}\right)-\frac{1}{m}\overset{m}{\underset{l=1}{\sum }}c_{i}^{\left[l\right]}\left(T_{2}\right)\right)}^{2}}, \end{array}$$

where *n* is the number of channels. *m* denotes the number of the network layers. $c_{i}^{\left[l\right]}\left(T_{1}\right)$ denotes the closeness centrality of the node *i* in the layer *l* of multilayer network *G*(*T*_1_). **C**(*T*_1_) is a matrix of size *n*×*m*, consisting of the node centralities of each node of each layer. The most sensitive parameters of the segmentation algorithm were set thusly: reference window length *W*_*r*_ = 2s, probability of outlier selection *p*_*KDE*_ = 0.96. Other parameter settings are the same as in our previous work (Xu et al., 2023).

**Figure 2:**
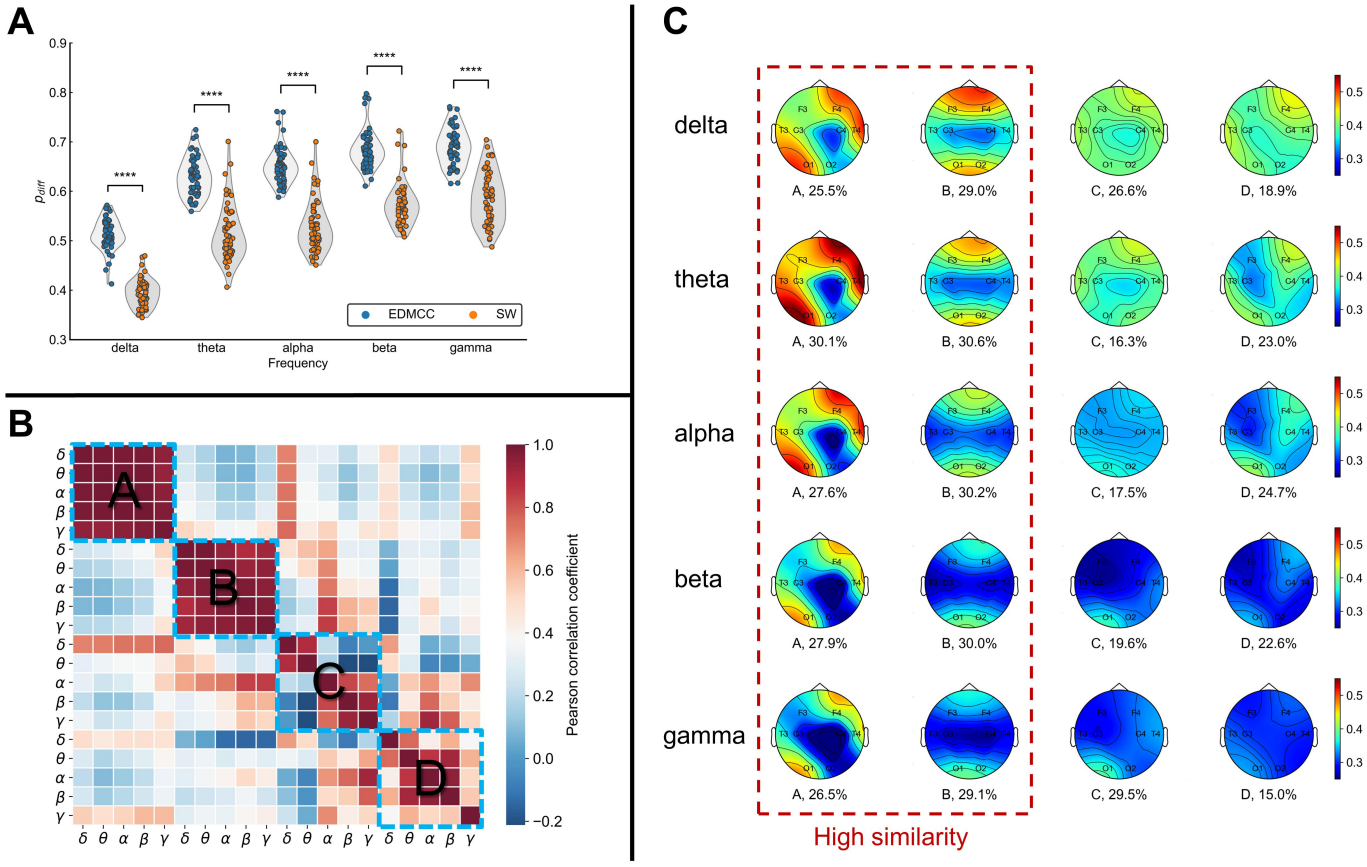
**(A)** Segmentation performance as measured with *p*_*diff*_ (higher values are better) for different frequency bands. EDMCC is an extension of our previous segmentation method to multilayer networks. SW denotes segmentation into same-length sliding windows. Each data point is the *p*_*diff*_ value of a block of EEG data in a frequency band. ****p < 0.0001. **(B)** Correlation of multilayer network states across states (ABCD) and frequency bands. **(C)** Average overlapping node closeness centrality of the multilayer networks for each multilayer network state (column). Numerical values under the topographic maps denote the ratio of total time spent in the network state.

Using this method, the EEG data were divided into segments of varying lengths (delta: 1.95 ± 0.835, theta: 1.998 ± 0.888, alpha: 2.07 ± 0.951, beta: 1.984 ± 0.843, gamma: 1.995 ± 0.859; average ± standard deviation seconds per segment). Compared with sliding windows of the same length (2 s for all bands), EDMCC performs better (see Figure 2A) in terms of an index that measures the difference in structure between segments compared to within segments [details on this *p*_*diff*_ index can be found in Xu et al. (2023)].

#### 2.2.3 Multilayer centrality metrics

We measure the structural characteristics of one multilayer network by calculating the node closeness centrality (CC) at each node and examine the differences between layers. CC quantifies the average length of the shortest path between the target node and all other nodes in the graph and encodes information about the information transmission latency across the network topology. For the multilayer network, we adopted the overlapping CC (referring to the concept of overlapping nodes across layers). CC of a node *i* is defined as:


(4) $$\begin{array}{l} CC_{i}=\frac{1}{m}\overset{m}{\underset{l=1}{\sum }}{\left(\frac{1}{n-1}\overset{n}{\underset{j\in N,j\neq i}{\sum }}d_{ij}\right)}^{-1}, \end{array}$$

where *N* is the set of all nodes in the network. *d*_*ij*_ is the length of the shortest path between node *i* and *j*. The distance between two adjacent nodes is defined as the inverse of the connection strength. The distance between layers was zero (unconstrained).

Then, we assessed the heterogeneity of a multilayer network by calculating the Pearson correlation between the edges in the different layers (see Figure 1D). A correlation value close to 1 indicates that the edges of the two layers are similar. A correlation value close to −1 indicates that the edge strengths of the two layers are in opposite directions. A correlation value close to 0 suggests that there is no linear relationship in the edges between the two layers.

### 2.3 Dynamic analysis of multilayer networks

#### 2.3.1 Defining network state

To define recurring network motifs across all participants and trials, we cluster networks based on the similarity of their structure. Here, we describe the method for one frequency band, and in practice we repeat this procedure (separately) over bands.

First, we calculate the overlapping node closeness centrality for each node in the multilayer network, shown in Figure 1E. Let the vector ${\text{c}}_{i,j,k}=\left(c_{i,j,k}^{\left(1\right)},c_{i,j,k}^{\left(2\right)},\ldots ,c_{i,j,k}^{\left(8\right)}\right),i=1,2,\ldots ,14,j=1,2,\ldots ,n_{i,k}$, *k*∈{*FL*−1, *FL*−2, *MW*−1, *MW*−2}, represent the overlapping node closeness centrality for the *i*-th participant from condition-trial *k* during time segment *t*_*j*_. The superscript of $c_{i,j,k}^{\left(l\right)}$ indicates the *l*-th channel in the EEG signal. *n*_*i, k*_ denotes the number of EEG segments for participant *i* in condition-trial *k*. To mitigate individual variability among participants, the vectors **c**_*i, j, k*_ of each participant are normalized as follows:


(5) $$\begin{array}{l} {\bar{c}}_{i,j,k}=\frac{c_{i,j,k}-\underset{j,k}{min}(c_{i,j,k})}{\underset{j,k}{max}(c_{i,j,k})-\underset{j,k}{min}(c_{i,j,k})}. \end{array}$$

Second, to perform clustering we construct a large-scale network (note, this is not a brain network, but a network for the clustering process) based on the multilayer network data from all participants and trials. Each multilayer brain network serves as a node here, and the edges represent the Spearman correlation coefficient of the normalized overlapping node closeness centrality between two networks. We use the Louvain algorithm for community detection on this large-scale network (Blondel et al., 2008). In this work, each community is referred to as a class or category and represents a recurring, typical multilayer network state (motif). We chose this clustering method because it does not require the pre-specification of meta parameters. The schematic diagram for this step is illustrated in Figure 1F.

Finally, the above steps are repeated for data in each frequency band. The overlapping node closeness centrality values in the multilayer networks of each class were separately (for different bands) averaged to obtain the visualization shown in Figure 2C.

The multilayer networks under each frequency band were ultimately clustered into four categories. From Figure 2C, we can see that the ratio of each category is relatively uniform (numerical values under the topographic map in Figure 2C). To assess the similarity levels between these categories and across frequency bands, we calculated the Pearson correlation *cor*(**c**_*ij*_, **c**_*ij*_) among states and bands. **c**_*ij*_ denotes the average (across instances of the state) overlapping node closeness centrality of the multilayer network state *i* in frequency *j*. The results are illustrated in Figure 2B. This analysis showed a notably higher correlation between frequencies for network states A and B, while states C and D had relatively lower correlations between frequency bands. This implies the emergence of more similar network structures for states A and B across all frequency bands. Conversely, states C and D show more disparities among frequencies.

Examining the topographic maps in Figure 2C, multilayer network state A displays a notably higher information transmission efficiency in the right frontal lobe and left occipital lobe compared to other brain areas. State B displays lower information transmission efficiency in the temporal lobes and central region compared to other brain areas. The topographic maps for states C and D show some differences across frequency bands, with an overall lower information transmission efficiency.

#### 2.3.2 Dynamic network characteristics

Subsequently, we assign EEG segments to network states to obtain a state sequence. If contiguous segments share the same network state, they are consolidated. This process yields a multilayer network state sequence for each trial (video, four per participant). A schematic diagram of state sequence construction is shown in Figure 1G. In order to characterize multilayer network state sequences, we use the following five metrics (Figure 1H):

(a) Frequency, the average number of occurrences per second of a given network state.
(b) Average duration, the average length of time a given network state remains whenever it appears.
(c) Coverage, computed as the time fraction of a given network state among the whole sequence.
(d) Transition probabilities of a network state X to a state Y, *P*_*X*→*Y*_. This is computed as the number of state transitions from state *X* to state *Y* divided by the total number of transitions in the whole sequence (also called observed transition probabilities).

In order to test the randomness of the multilayer network state transitions, we performed a permutation test. The null hypothesis assumes no association between the current state and the next state. Consequently, the expected transition probability (due solely to frequencies) is defined as:


(6) $$\begin{array}{l} P_{X\to Y}^{*}=\frac{P_{Y}\cdot P_{X}}{1-P_{X}}, \end{array}$$

where *P*_*X*_(*P*_*Y*_) is the number of occurrences of state *X*(*Y*) divided by the number of all states observed. For each condition, we calculated the average observed transition probabilities and the average expected transition probabilities. Then, the average observed transition probabilities and average expected transition probabilities are randomly permutated *n* times. The distance for each permutation is computed using the formula below:


(7) $$\begin{array}{l} d=\underset{X,Y}{\sum }\frac{{\left(P_{X\to Y}-P_{X\to Y}^{*}\right)}^{2}}{P_{X\to Y}^{*}}. \end{array}$$

The actual chi-square distance is the difference between the average expected transition probability and the average observed transition probability without permutation. Let *m* denote the count of instances where the chi-square distance of random permutation exceeds the actual chi-square distance. The *p*-value is then *p* = *m*/*n*. If the *p*-value is < 0.05, we reject the null hypothesis. This means that there is structure in the observed transition values that cannot be attributed solely to the frequencies of the states.

### 2.4 Statistical analysis

In our statistical tests, unless explicitly stated otherwise, we consistently adhere to the following procedure. We use Tukey's box plot method for the removal of outlier data points (Tukey et al., 1977), defined as values exceeding 1.5 times the interquartile range (IQR) above the upper quartile or below the lower quartile. Subsequently, we assess the normality assumption of the data. If normality is not rejected, we proceed to Levene's test for equality of variances. Upon passing Levene's test, we employ *t*-tests with equal variance; otherwise, we use *t*-tests with non-equal variance. In instances where the normality assumption is rejected, the rank-sum test is used. Our significance threshold is set at 0.05. We present raw *p*-values and significance results, without correction for multiple comparisons; thus one should expect about 1 in 20 of significant effects to be spurious.

### 2.5 Classification

**Figure 3:**
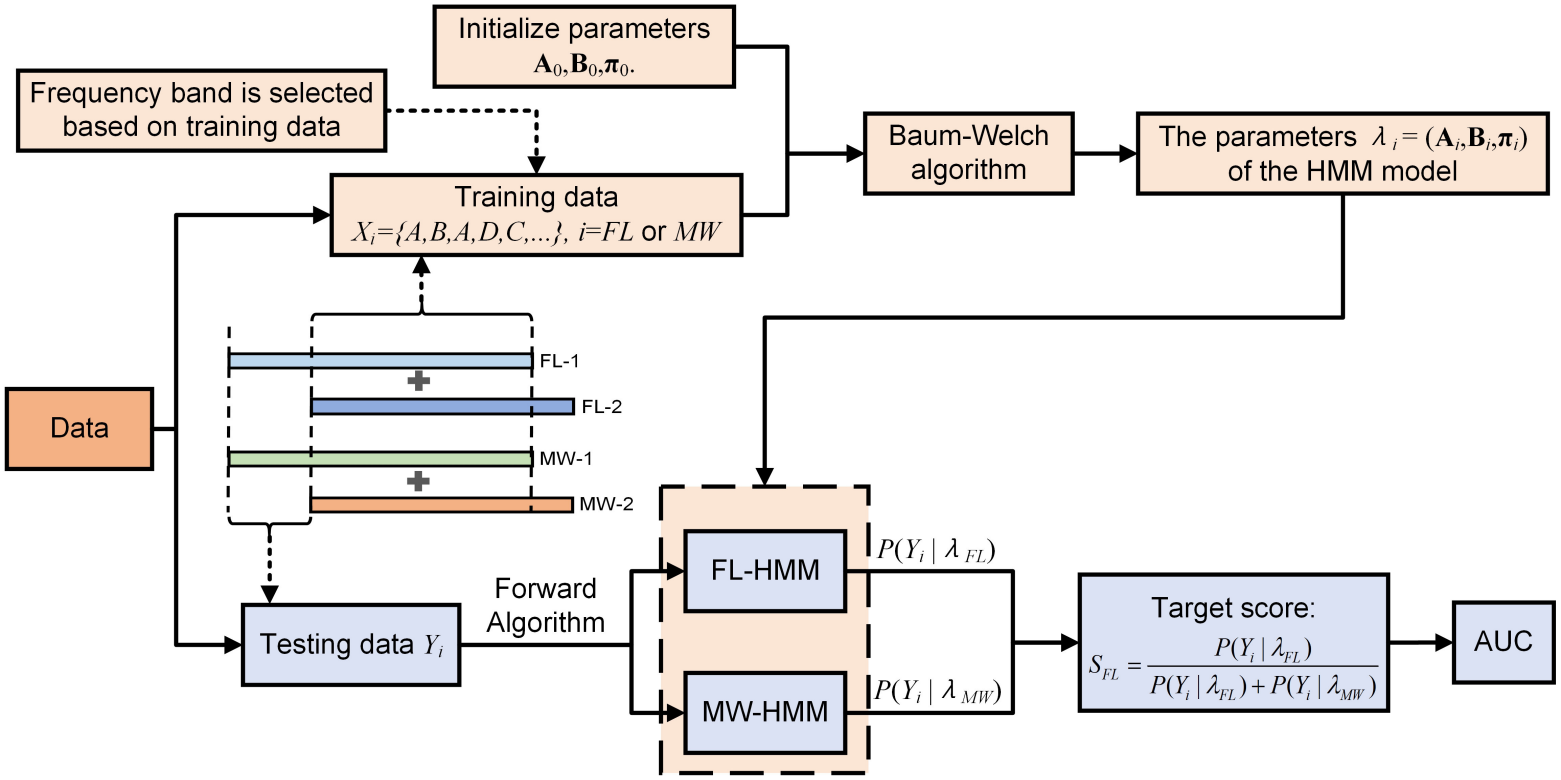
Flowchart of our method for detection of mind-wandering via HMM.

The observed distinctions in the multilayer network state sequences between the MW and FL conditions suggest the potential applicability of these sequences for the detection of MW. To substantiate the efficacy of this approach, we conducted perparticipant classification using a hidden Markov model (HMM) based classifier. The schematic diagram for this step is showed in Figure 1I. The HMM takes as input the discretized state sequence (with four possible observation classes). The decoding process is illustrated in Figure 3. The algorithmic procedure is outlined below:

1. We select one participant from a pool of 14 individuals in turn. We obtain multilayer network state sequences for the chosen participant for each of the five frequency bands. For each frequency band, four sequences are obtained, one per trial (video).
2. Concatenating state sequences across trials of the same condition, we perform 8-fold cross-validation to partition the data into training sets and test sets.
3. We fit parameters (steps iv–vi below) using the training set of the fold. We choose the frequency band based only on classification performance in the training set of a cross-validation fold.
4. Using the Baum-Welch algorithm (Baggenstoss, 2001), the entire training set for one condition is treated as one sequence (by concatenation) to construct an HMM in an unsupervised manner. We set the number of hidden states to 2. The initial transition probabilities of hidden states **A**, initial observation probabilities **B**, and the initial probabilities for hidden states **π** are all set to be uniformly distributed. We obtain two HMMs, one for MW and one for FL.

5. Each multilayer network state sequence (trial) of the test set is given to the forward algorithm along with either the MW HMM or FL HMM obtained in step iv. The forward algorithm calculates the likelihood *P*(*O*|λ_*i*_) associated with each condition's HMM. Assuming a uniform prior, the posterior probability (which we use as the score of the model) is just the normalization of the likelihood:
(8) $$\begin{array}{l} S_{FL}=\frac{P\left(Y_{i}|\lambda_{FL}\right)}{P\left(Y_{i}|\lambda_{FL}\right)+P\left(Y_{i}|\lambda_{MW}\right)}, \end{array}$$
  where *Y*_*i*_ denotes the observed test sequence, while λ_*i*_ represents the estimated parameters (**A**_*i*_, **B**_*i*_, π_*i*_) of the HMM model for state *i*, where *i* is FL or MW.
6. The classification result is decided by thresholding the score *S*_*FL*_, where the threshold is set in the receiver operating characteristic calculation procedure. We quantify classification performance by the area under the receiver operating characteristic curve (AUC).
7. We repeat steps i–vi for each fold in the cross-validation, and then average the AUC across folds. We then repeat all steps for each participant.

## 3 Results

### 3.1 Static analysis results

First, we conducted statistical analysis on a multilayer network created from EEG data from all trials (of the same condition) of all 14 participants. Since one network was made from the data in the entire trial, this is a static analysis. We examined the edges, nodes, and inter-layer heterogeneity under mind-wandering or focused learning.

**Figure 4:**
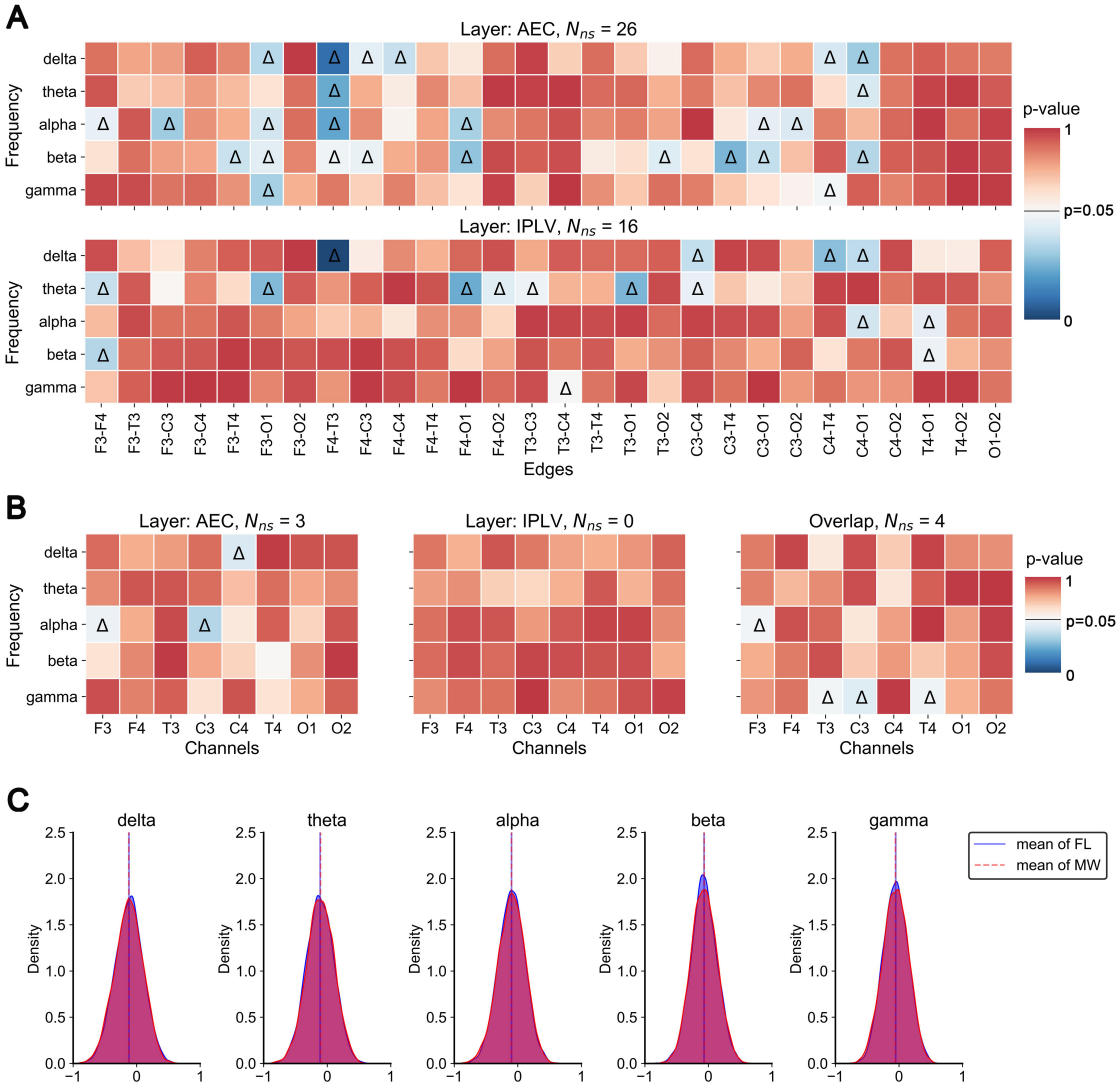
Static analysis results. **(A)** *P*-value plot of significant differences in edge weights between MW and FL. Color in the heat map indicates the *p*-value of the statistical test. Triangles indicate significant differences. **(B)** *P*-value plot of significant differences in node closeness centrality between MW and FL. **(C)** Histograms (across participants and trials) of Pearson correlation values between corresponding edges of the two layers.

We found significant differences in intra-layer connections within the multilayer network across frequency bands (Figure 4A). In the AEC layer, significant differences (indicated by triangles) were observed in the edges F3-O1 and F4-T3 across the four frequency bands. Significant differences in edges were mainly concentrated in the delta, alpha, and beta frequency bands. In the IPLV layer, significant differences in edges were mainly concentrated in the theta band.

We found three significant differences in the node closeness centrality (single layer) of the AEC layer in the delta and alpha bands (Figure 4B). However, we found no significant difference in the node closeness centrality in the IPLV layer. We found four significant differences in the overlapping node closeness centrality (entire multilayer network) in the alpha and gamma frequency bands.

Finally, we examined inter-layer heterogeneity via Pearson correlation of intra-layer connections (for each participant). This distribution of correlation coefficients (among participants and trials) is presented in Figure 4C. The analysis results indicate that the majority of inter-layer correlation coefficients are relatively low, predominantly concentrated around 0. This means there was not much in common in the structure between the layers.

### 3.2 Dynamic analysis results

**Figure 5:**
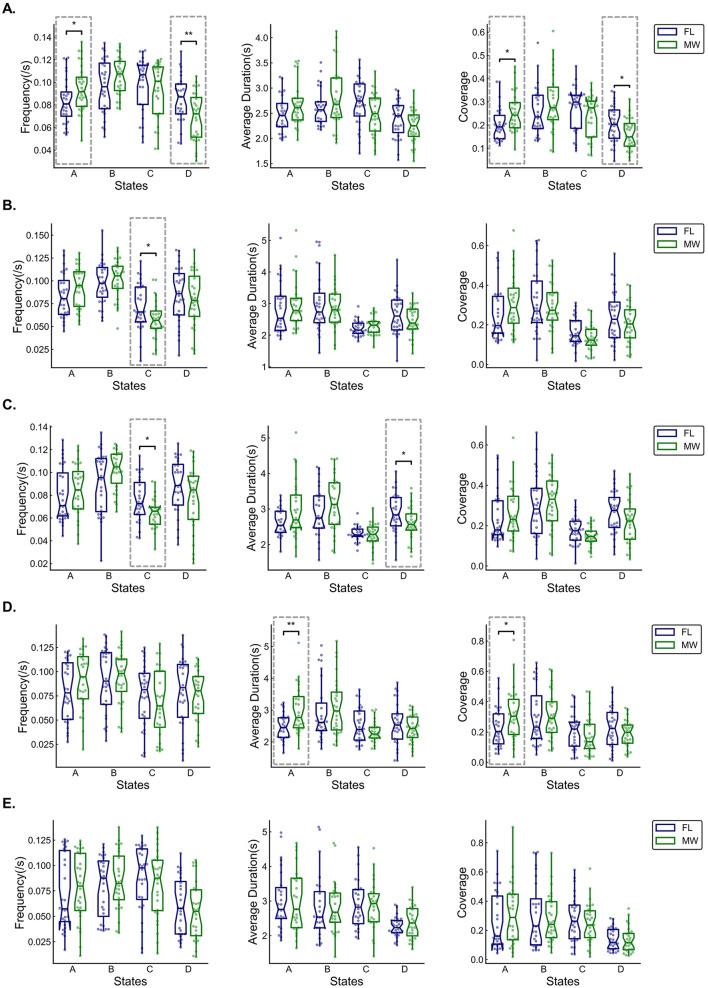
Box and whisker plots (across participants) of occurrence frequency, average duration, and coverage of each multilayer network state for delta **(A)**, theta **(B)**, alpha **(C)**, beta **(D)**, and gamma **(E)** frequency bands. Statistical significance (dashed boxes) was set at 0.05. *p < 0.05, **p < 0.01, ***p < 0.001.

**Figure 6:**
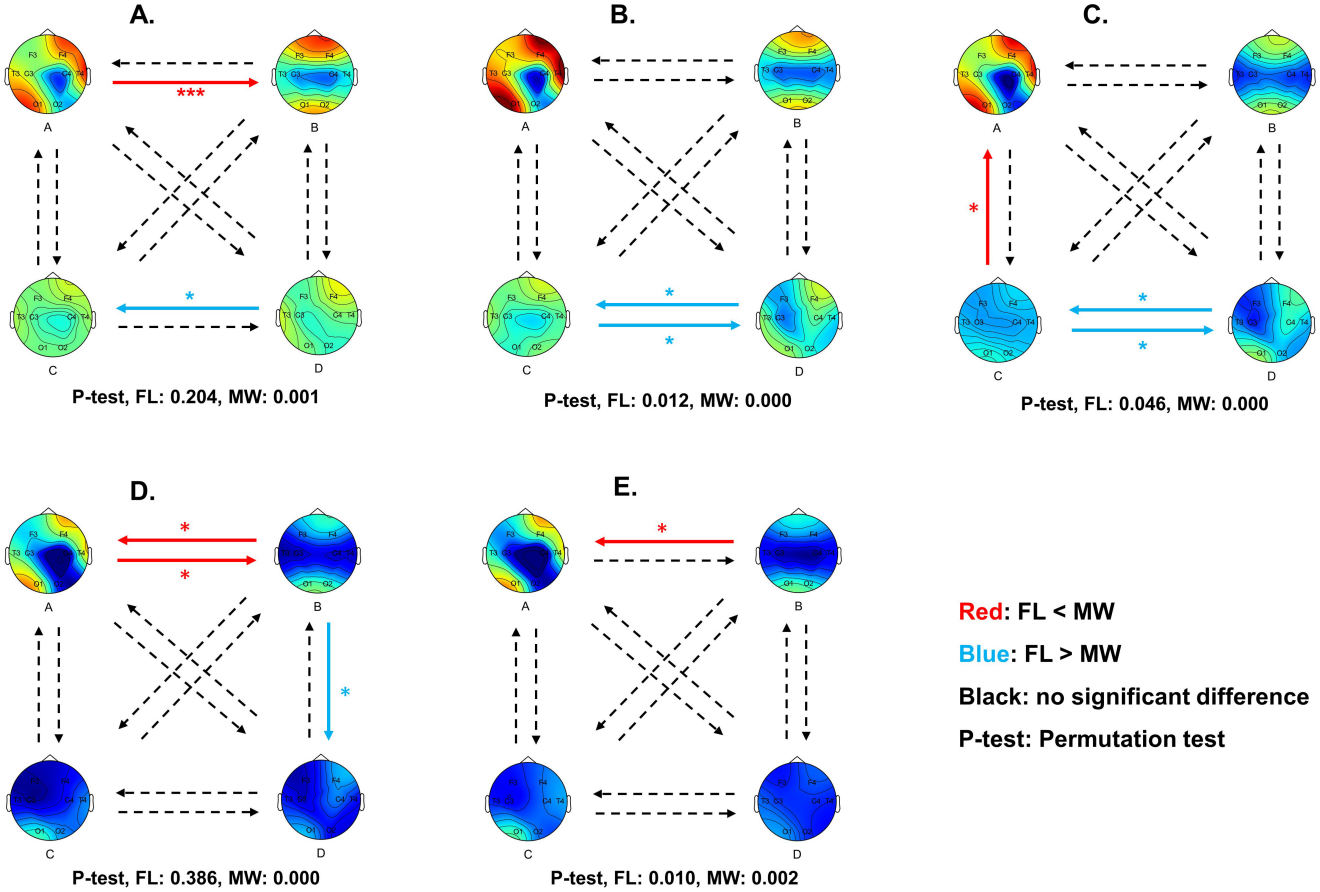
Diagram of multilayer network state transition probability differences between MW and FL for delta **(A)**, theta **(B)**, alpha **(C)**, beta **(D)**, and gamma **(E)** frequency bands. Red arrows indicate a significantly higher transition probability in MW compared to FL, while blue arrows indicate a significantly lower transition probability. Statistical significance was set at 0.05. *p < 0.05, **p < 0.01, ***p < 0.001.

We examined differences in the dynamic features computed from the multilayer network state sequences during FL vs. MW, pooling data across participants-trials (Figures 5, 6).

For the delta frequency band (Figure 5A), MW had a significantly higher occurrence frequency (*p* = 0.042) for the multilayer network state A. MW had significantly lower the state D frequency (*p* = 0.009), state A coverage (*p* = 0.028), and state D coverage (*p* = 0.024). As shown in Figure 6A, the transition probability from state A to state B was significantly higher for FL (*p* = 0.000). The transition probability from state D to state C was significantly lower for FL (*p* = 0.011). The permutation test checking for fully random transitions yielded *p*=0.204 for FL and *p* = 0.001 for MW. This means that there was structure in the observed transition values (for states found in our analysis) in the MW condition that cannot be completely attributed to the frequencies of the states.

For the theta frequency band (Figure 5B), MW had significantly lower state C frequency (*p* = 0.026). As shown in Figure 6B, the transition probabilities from state C to state D (*p* = 0.012) and from state D to state C (*p* = 0.029) were significantly lower for FL. The permutation test yielded *p* = 0.012 for FL and *p* = 0.000 for MW. This means that there was structure in the observed transition values for both conditions that cannot be completely attributed to the frequencies of the states.

For the alpha frequency band (Figure 5C), MW had significantly lower state C frequency (*p* = 0.028) and state B average duration (*p* = 0.04). As shown in Figure 6C, the transition probabilities from state C to state D (*p* = 0.038) and from state D to state C (*p* = 0.026) were significantly lower for FL. The transition probability from state A to state B (*p* = 0.039) was significantly higher for FL. The permutation test yielded *p* = 0.046 for FL and *p* = 0.000 for MW. This means that there was structure in the observed transition values for both conditions that cannot be completely attributed to the frequencies of the states.

For the beta frequency band (Figure 5D), MW had significantly higher state A average duration (*p* = 0.003) and state A coverage (*p* = 0.047). As shown in Figure 6D, the transition probabilities from state A to state B (*p* = 0.015) and from state B to state A (*p* = 0.036) were significantly higher for FL. The transition probability from state B to state D (*p* = 0.030) was significantly lower for FL. The permutation test yielded *p* = 0.386 for FL and *p* = 0.000 for MW. This means that there was structure in the observed transition values in the MW condition that cannot be completely attributed to the frequencies of the states.

For the gamma frequency band (Figure 5E), there were no significant differences between FL and MW in the dynamics characteristics. As shown in Figure 6E, the transition probability from state B to state A (*p* = 0.038) was significantly higher for FL. The permutation test yielded *p* = 0.010 for FL and *p* = 0.002 for MW. This means that there was structure in the observed transition values for both conditions that cannot be completely attributed to the frequencies of the states.

In terms of frequency bands, delta had more pronounced differences between MW and FL, indicating a closer association between the delta frequency band and MW. In terms of states, there was a higher occurrence of significant differences for states A and B across frequency bands, with state A exhibiting more significant differences, suggesting its relative importance in MW.

### 3.3 Classification performance

**Figure 7:**
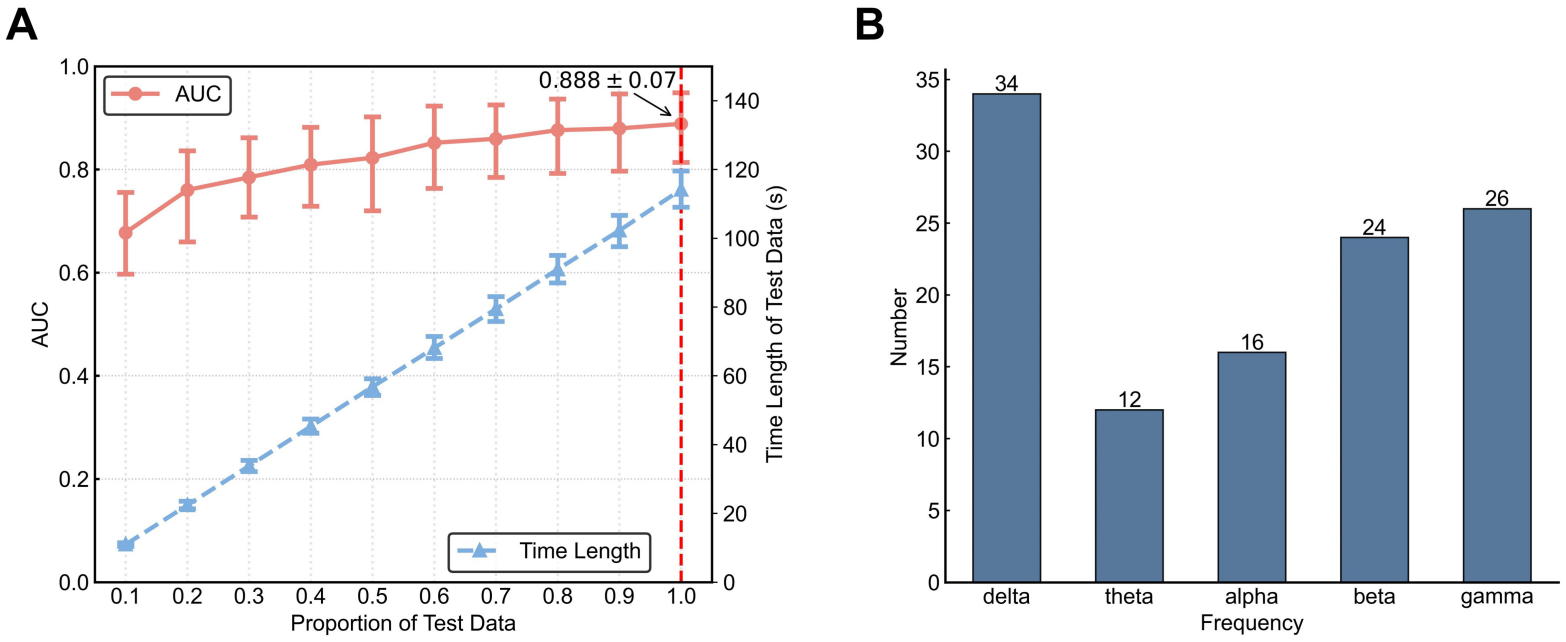
Decoding results using an HMM-based classifier. **(A)** AUC for different test set lengths. Left vertical axis for AUC corresponds to the red line. Error bars represent 95% confidence intervals. Right vertical axis for testing set length corresponds to the blue line. Horizontal axis denotes proportion of the testing set. **(B)** Number of times each frequency band was automatically chosen during parameter fitting.

We used state sequences as input to a HMM based method for classification of MW vs. FL. The mean (across participants) classification AUC was 0.888 ± 0.070 [mean ± standard deviation(std)]. We examined how dependent classification accuracy was on the length of a sequence by varying the proportion of the entire testing data sequence used (Figure 7A). Results indicate that with an increasing amount of testing data, the accuracy rises: this increase is fast for less than about 25 s of data and slower for more than 25 seconds of data. Considering that our decoding algorithm automatically selects frequency band based on the training data, we conducted a statistical analysis of the frequency band selection results. From Figure 7B, it is evident that the delta frequency band was selected most often. This is consistent with our above findings in terms of dynamics feature differences, suggesting that delta band has the most useful features. Using the experimental data examined in this work, Tang et al. employed a radial basis function kernel support vector machine classifier with Riemannian-processed covariance features. Their approach had a mean AUC of 0.876 ± 0.070 for within-participant prediction (Tang et al., 2023).

To compare the classification results from the HMM with traditional classification methods, we used band-power features of EEG signals across various frequency bands and static functional connectivity features (AEC and IPLV were chosen as functional connectivity measures because they were used to calculate the multilayer network connections in this study) as inputs to machine learning models, including radial basis function kernel support vector machine (SVM), logistic regression (LR), and decision tree (DT). The parameters of these classifiers were the default values of the scikit-learn (version 0.24.1) implementations we used. For these comparison methods, we report the best classification results among all frequency bands. Note that these traditional methods employed the same 8-fold cross-validation protocol as the HMM algorithm. The time segments corresponding to the training and testing sets were the same as those for the HMM classification. Furthermore, to provide a more comprehensive assessment of classification performance, we also report the F1 score, as detailed in Table 1. The classification results showed that HMM performed better than the comparison methods. The primary aim of this study is to show that dynamic brain network analysis can capture differences in brain activity during periods of MW vs. focused learning. The classification performance of the HMM using dynamics analysis features further supports the effectiveness of dynamics analysis for detecting MW.

**Table 1:** Results of mind-wandering detection by different classification methods.

**Classification methods**		**AUC (mean ± std)**	**F1 score (mean ± std)**
HMM		0.888 ± 0.070	0.851 ± 0.107
(Tang et al., 2023)		0.876 ± 0.070	–
Power	SVM	0.851 ± 0.188	0.802 ± 0.185
	LR	0.876 ± 0.122	0.774 ± 0.174
	DT	0.795 ± 0.139	0.796 ± 0.142
AEC	SVM	0.870 ± 0.123	0.817 ± 0.167
	LR	0.885 ± 0.104	0.817 ± 0.167
	DT	0.804 ± 0.147	0.807 ± 0.144
IPLV	SVM	0.551 ± 0.086	0.533 ± 0.137
	LR	0.556 ± 0.108	0.711 ± 0.108
	DT	0.709 ± 0.202	0.750 ± 0.172

## 4 Discussion

We proposed a network dynamics analysis method for EEG data based on multilayer functional connectivity networks and a classification method using network state sequences as input for detecting mind-wandering states. In our work, four typical multilayer network states were found. Two of these states were similar between frequency bands. We found that statistics of the multilayer network state sequence (network dynamics features) were significantly different between MW and focused learning. Finally, when we used the multilayer network state sequence as input for an HMM-based classifier, we could detect MW with 0.888 AUC (within-participant prediction).

While there are limitations on the interpretation of network analysis when using EEG electrodes as nodes, instead of using voxels or regions of interest as nodes, due to the possible confound of volume conduction effects, we have mitigated this to some extent by choosing AEC and IPLV as the connectivity metrics, which are known to be relatively less sensitive to volume conduction effects (Lai et al., 2018; Jian et al., 2017). Still, caution is advised in the neuroscientific interpretation of our results.

In comparison to the FL condition, the state A during MW exhibits significantly higher occurrence frequency, average duration, and coverage in the delta and beta frequency bands (Figures 5A, D). In addition, state D shows significantly lower frequency, average duration, and coverage in the delta and alpha frequency bands (Figures 5A, C). The state A is characterized by stronger information transfer in the frontal lobe area and left occipital lobe area compared to other brain regions. The multilayer network state D is characterized by weaker information transfer in the left frontal lobe area compared to other brain regions. This suggests that the efficiency of information transmission in the frontal lobe area and left occipital lobe area is higher during MW.

Previous studies indicate a correlation between the prefrontal cortex activity and MW (Bernhardt et al., 2014; Chou et al., 2017). Godwin et al. (2023) found that, compared to on-task and task-related interference, during off-task and inattention states, there is higher fMRI activation within the inferior frontal gyrus. Braboszcz and Delorme (2011) found that occipital and fronto-lateral power was significantly changed in the MW state compared to the breath focus state. Through EEG recordings during live lectures, Dhindsa et al. (2019) observed significant changes in power across occipitoparietal, frontal, temporal, and occipital regions. To our knowledge, there is little research on the dynamics of functional connectivity networks of MW. The findings of our study offer a basis for future research.

EEG MW detection studies have predominantly focused on event-related potentials (ERP) and spectral features. Dong et al. (2021) classified attention states both within and across participant using ERP measures and support vector machines, obtaining 0.715 AUC within participant and 0.613 AUC across participants. Dhindsa et al. (2019) used common spatial patterns to discover scalp topologies for individual-level classification of MW, obtaining average accuracy of 80–83%. Kawashima and Kumano (2017) used power and coherence of EEG signals to detect MW during sustained attention to response tasks. Their findings suggest that a nonlinear model incorporating multiple electrodes exhibited higher predictive accuracy for MW compared to a linear model using individual electrodes. Tang et al. (2023) employed spatial covariance features, processed through Riemannian geometry (using the same EEG data as in this study) with a radial basis function kernel support vector machine classifier. Unlike the above, in this study, we perform MW detection using multilayer network state sequences as input for an HMM classifier, offering a promising new feature construction methodology.

The analysis and results in this study are based on EEG data from 14 participants, which is relatively small and a limitation of our work. In the future, we hope to include more participants to further validate results. While EEG offers superior temporal resolution compared to fMRI, its spatial resolution is relatively limited. Since the current study is exploratory in nature, many results can benefit from verification. Further studies with more channels and experimental data can help confirm our analyses and investigate the characteristics of network dynamics under MW. Notably, our dataset has only eight channels, a deliberate choice aimed at evaluating methods that are feasible when paired with a practical recording system. We intend to increase the number of channels in forthcoming experiments and analyses. The electrode-level functional connectivity in this study is susceptible to volume conduction, implying that a portion of the computed connectivity may originate from physics rather than neural synchrony. To address this, our future work will incorporate source localization, facilitating network analysis in terms of brain regions and mitigating volume conduction effects.

## 5 Conclusion

We investigated the dynamical characteristics of multilayer functional connectivity networks during mind-wandering. Our findings reveal relationships between the dynamical changes in multilayer networks and mind-wandering. By using multilayer network state sequences as input features, we were able to detect participants' mind-wandering. Our approach demonstrates the potential to analyze the dynamics of multilayer functional connectivity networks constructed from EEG data and detect mind-wandering based on analysis results.

## Data Availability

Publicly available datasets were analyzed in this study. This data can be found here: https://osf.io/7mj5e.
